# Microsomal triglyceride transfer protein in the ectoparasitic crustacean salmon louse (*Lepeophtheirus salmonis*)[S]

**DOI:** 10.1194/jlr.M076430

**Published:** 2017-06-10

**Authors:** Muhammad Tanveer Khan, Sussie Dalvin, Frank Nilsen, Rune Male

**Affiliations:** Departments of Biology* University of Bergen, N-5020 Bergen, Norway; Molecular Biology,§ Sea Lice Research Centre, University of Bergen, N-5020 Bergen, Norway; Sea Lice Research Centre,† Institute of Marine Research, 5817 Bergen, Norway

**Keywords:** lipid transport proteins, lipid transport, gene expression, lipid and lipoprotein metabolism, lipoproteins, RNA interference, Nile Red, Oil Red O, sea lice

## Abstract

The salmon louse, *Lepeophtheirus salmonis*, is an endemic ectoparasite on salmonid fish that is challenging for the salmon farming industry and wild fish. Salmon lice produce high numbers of offspring, necessitating sequestration of large amounts of lipids into growing oocytes as a major energy source for larvae, most probably mediated by lipoproteins. The microsomal triglyceride transfer protein (MTP) is essential for the assembly of lipoproteins. Salmon lice have three *L. salmonis* MTP (*LsMTP*) transcript variants encoding two different protein isoforms, which are predicted to contain three β-sheets (N, C, and A) and a central helical domain, similar to MTPs from other species. In adult females, the LsMTPs are differently transcribed in the sub-cuticular tissues, the intestine, the ovary, and in the mature eggs. RNA interference-mediated knockdown of *LsMTP* in mature females gave offspring with significantly fewer neutral lipids in their yolk and only 10–30% survival. The present study suggests the importance of LsMTP in reproduction and lipid metabolism in adult female *L. salmonis*, a possible metabolic bottleneck that could be exploited for the development of new anti-parasitic treatment methods.

The microsomal triglyceride transfer protein (MTP) was first reported as an endoplasmic reticulum resident protein that catalyzes the transfer of neutral lipids between membranes (1). Later it was found that MTP is also essential for the synthesis and secretion of lipoproteins containing apoB (2). MTP belongs to the large lipid transfer protein superfamily and it functions as a transporter of lipids in the assembly of nascent lipoprotein particles within the endoplasmic reticulum (3). This protein family also contains other members with a central role in animal reproduction and lipid circulation, such as vitellogenins, vertebrate’s apoB, and insect apolipophorin (apoLp)-II/I (4, 5).

MTP is a heterodimeric protein complex composed of two distinct subunits, a large subunit of, typically, 99 kDa containing a lipid transfer activity and a multifunctional 58 kDa protein disulfide isomerase (PDI) (6, 7). The MTP large subunit, for simplicity named only MTP, is composed of three structural motifs: the N-terminal β-barrel (N-sheet), the central α-helix domain and C-terminal β-sheet (C-sheet), and three functional domains (lipid transfer, membrane-associating, and apoB binding) (8, 9). The N-sheet β-barrel is involved in the recognition of the N terminus of apoB, the central α-helix interacts with both apoB and PDI, and the C-sheet β-sheet has lipid binding as well as transfer properties (8).

In mammals, MTP is essential for the assembly and secretion of apoB-containing lipoprotein, chylomicrons in the intestine, and VLDLs in the liver (10), and thereby facilitates delivery of triglyceride and cholesterol to the peripheral tissues. In humans, homozygous mutations in the *MTP* gene abolish secretion of apoB-lipoproteins and reduce the lipid level in plasma, resulting in abetalipoproteinemia (11). Studies in the mice show that homozygous knockout of the *MTP* gene is lethal to the embryo (12). This phenotype is ascribed to the lack of lipoprotein synthesis and massive accumulation of lipid droplets in the cells of yolk sac endoderm (12), indicating that the yolk sac has lost its ability to produce lipoproteins and deliver lipids to the developing embryo.

In oviparous organisms, such as frog (13), *Drosophila* (14, 15), and worms (16), MTP has a similar function in the secretion of lipoproteins as found in mammals. The secretion of frog vitellogenin A-1 is MTP dependent (13), as the secretion is enhanced only when coexpressed with MTP. In *Drosophila melanogaster*, the transport of lipids between organs is carried out by a single apoB-family lipoprotein, lipophorin (Lpp) (14). The lipidation of Lpp occurs in two continuous steps with the help of two distinct lipid transfer proteins, MTP and large lipid transfer particle. Initially, Lpp is released from the fat body as a phospholipid-rich particle through the MTP-dependent mechanism and reaches the gut, where it is loaded with sterols and diacylglycerols via large lipid transfer particle. A homolog of the large subunit of MTP, named defecation suppressor of Clk (DSC-4), was found in the intestine of *Caenorhabditis elegans* (16).

The salmon louse (*Lepeophtheirus salmonis*) is a marine ectoparasitic copepod that infests salmonids in the Northern Hemisphere. The salmon louse feeds on the blood, mucus, and skin of hosts and represents a major health and fish welfare issue that causes large economic losses in the Atlantic salmon (*Salmo salar*) farming industry (17) and also poses a considerable threat to wild salmonids (18). The lifecycle of the salmon louse consists, in total, of eight stages, each separated by a molt (19). The first are two stages of free-living nauplius larva followed by one infective copepodid stage. This is followed by two chalimus stages (where the parasites are firmly attached to the host), two preadult stages (with clear morphological sex difference), and finally the adult stage. Before host attachment, larvae are lecithotrophic, dependent on energy from maternally deposited lipid and protein reserves within the yolk (20, 21). The sexually mature adult female continuously produces eggs carried in two egg-strings. Under laboratory conditions, female salmon lice can survive for at least 455 days and produce more than 11 pairs of egg-strings (22). During egg production, the female louse incorporates massive amounts of yolk proteins (21, 23) and lipids into the growing oocytes. The predominant lipids in the eggs are neutral lipids, triacylglycerol, and cholesterol, followed by polar lipids, such as phosphatidylcholine and phosphatidylethanolamine, but fatty acid composition varies with the composition of the food received by the host salmon (20). The mechanism of lipid accumulation in the growing oocytes has not been described in salmon lice. However, lipids are absorbed in the intestine and are likely to be transported with the hemolymph via lipoproteins, for example, to the oocytes. A highly efficient lipid uptake and transport can be predicted to secure the high production of eggs in salmon lice and dispersal of louse larvae in the environment.

In the present study, we identified a gene encoding MTP from *L. salmonis* (*LsMTP*). We hypothesize that LsMTP may be involved in the lipoprotein-based supply of lipids from the intestine of a female salmon louse to growing oocytes. To this end, *LsMTP* was characterized and three transcript variants were identified. Silencing of the *LsMTP* gene using RNA interference (RNAi) affected production of eggs and reduced the viability of the developing larvae due to less neutral lipids in their yolk. Our results suggest that LsMTP has a crucial role in the reproduction of female salmon lice.

## MATERIALS AND METHODS

### Sampling of salmon lice

A laboratory strain of salmon lice, *L. salmonis* (22), was kept on Atlantic salmon (*Salmo salar*) in tanks with a continuous supply of seawater (temperature 10°C and salinity 34.5 ppt). Fish were fed a commercial diet daily. Nauplii I/II and copepodids were obtained from hatching egg-strings in hatching incubators supplied with the same seawater. Chalimus, preadult, and adult stages of lice were sampled from fish. Prior to sampling, fish were anesthetized with a mixture of benzocaine (60 mg/l) and metomidate (5 mg/l) in seawater. All the experiments and maintenance of salmon were carried out according to the Norwegian animal welfare legislation.

For stage-specific quantitative (Q)-PCR, five biological replicates were collected from each stage. The following life stages and number of animals were collected for each replicate. Nauplius I (n = 100), nauplius II (n = 100), planktonic copepodid (n = 100), chalimus I (n = 10), chalimus II (n = 10), preadult I male and female (n = 1), preadult II male and female (n = 1), adult male (n = 1), young adult female and adult female (n = 1). For the starvation experiment, adult female lice were collected from fish and kept in seawater for 1–4 days. All the samples were stored in RNAlater^TM^ (Ambion) and kept overnight at 4°C prior to storing at −20°C for further use.

### RNA isolation and cDNA synthesis

Total RNA was extracted using TRI-reagent (Sigma-Aldrich) according to the manufacturer’s instructions. The concentration and purity of isolated RNA was confirmed using Nanodrop ND-1000 spectrophotometer (NanoDrop Technologies). The isolated total RNA samples were treated with amplification grade DNaseI (Invitrogen) as per manufacturer’s instructions. For Q-PCR, DNase-treated total RNA (250 ng) was used for cDNA synthesis with Affinity Script QPCR cDNA synthesis kit (Stratagene) and diluted 10 times with nuclease-free water prior to storage at −20°C. For PCR, 1 μg total RNA was reverse transcribed using a qScript cDNA SuperMix (Quanta Bioscience).

### Genome analysis, PCR, cloning, and sequencing of *LsMTP* gene

*LsMTP*-coding sequence was identified in the Ensembl database (http://r9ywwtvj.ensemblgenomes.org/Lepeophtheirus_salmonis/Info/Index) and the salmon louse genome database (accession: EMLSAT00000001530) (LiceBase, https://licebase.org/) with homology to known human (NCBI: X91148.1) and *Drosophila* MTP (FlyBase: FBgn0266369). The GenBank accession numbers of the three MTP sequences reported here are: LsMTP-A, MF063064; LsMTP-B, MF063065; and LsMTP-C, MF063066. PCR was carried out using GoTaq Flexi DNA polymerase (Promega) as per the manufacturer’s protocol. The 5′ and 3′ rapid amplification of cDNA ends (RACE) was conducted with SMARTer RACE cDNA amplification kit (Clontech) as instructed in the users’ manual. The 5′ and 3′ RACE-Ready cDNAs were synthesized from the total RNA of adult females and used for RACE-PCR. Gene-specific primers for 5′ and 3′ RACE are listed in supplemental Table S1. PCR products were cloned into pCR™ 4-TOPO® vector using the TOPO TA cloning kit for sequencing (Life Technologies) followed by transformation into *Escherichia coli* TOP10 cells. Clones were verified by PCR with M13 forward and reverse primers (supplemental Table S1). PCR products of positive clones were cleaned with ExoSAP-it (Affymetrix) and sequenced using BigDye Terminator v3.1 reagent (Applied Biosystems) at the sequencing facility of the University of Bergen.

### In situ hybridization

**Fig. 1.**
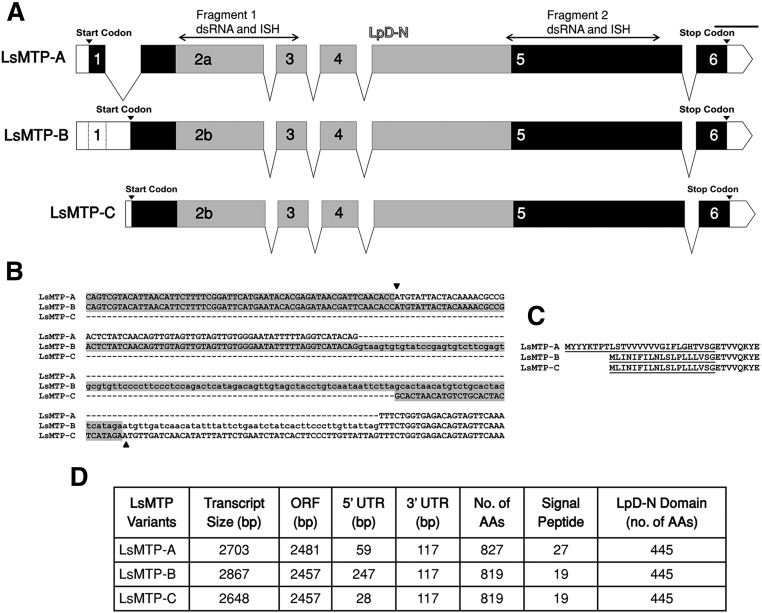
The organization of the *LsMTP* gene. A: Genetic structure of the three variants of *LsMTP*. *LsMTP-A* consisted of six exons with an initiator codon in exon 1. The 5′ UTR is represented with a white box. *LsMTP-B* was generated due to intron retention, with exon 1 as part of the 5′ UTR. *LsMTP-C* arose due to exon 1 skipping. Lipoprotein N-terminal domain (LpD-N) is shaded with gray. The positions of the fragments used for RNAi and in situ hybridization RNA probes (ISH) are also shown. Scale bar = 200 bp. B: Multiple nucleotide alignments of the 5′ UTR sequences of three *LsMTP* variants. The 5′ UTR nucleotide sequences of three variants are highlighted in gray. The arrowheads indicate the start codon (ATG). Lowercase letters represent the intron sequence. Gaps are displayed as dashed lines. C: N-terminal amino acid sequence alignment of three LsMTP variants. The predicted signal peptides for three variants are underlined. D: Overview table. The table lists the size of the variants, open reading frame (ORF), 5′ and 3′ UTRs, signal peptide, and LpD-N domain size in number of amino acids (AAs).

To confirm the in situ hybridization specificity, two different single stranded digoxigenin (DIG)-labeled RNA probes of 476 bp and 604 bp lengths corresponding to different regions of LsMTP transcripts (Fig. 1) were synthesized separately from cDNA using the DIG RNA labeling kit (Roche). Primers used for the synthesis of sense and antisense RNA probes are listed in supplemental Table S1. The concentration and labeling efficiency of probes was assessed by spectrometry (Nanodrop ND-1000) and with a spot test on nylon membrane, respectively. In situ hybridization was carried out in paraffin-embedded sections of adult female lice, as previously described by Kvamme, Frost, and Nilsen (24) and Dalvin, Nilsen, and Skern-Mauritzen (25) with some modifications. Tissue sections were deparaffinized with Histoclear (National Diagnostic) instead of xylene and proteinase K treatment was done for 13 min. Hybridization of probes (500 ng/100 μl) was carried out at 65°C for 16–20 h. Sections were incubated with anti-DIG-alkaline phosphatase Fab fragments (Roche) and visualized using nitroblue tetrazolium (Roche) and 5-bromo-4-chloro-3-indolyl phosphate (Roche). The localization of *LsMTP* transcripts was detected with antisense probes and sense probes were used as negative controls.

### Real-time Q-PCR

Q-PCR was performed on Applied Biosystem 7500 real-time PCR system using PowerUp SYBR Green Master Mix (Applied Biosystem) as per the manufacturer’s recommendations. The Primers used in Q-PCR are listed in supplemental Table S1. The salmon louse elongation factor 1α (ef1α) was used as a reference (26). Two-fold serial dilutions (six dilutions) of cDNA were used to create a standard curve for efficiency calculation. As the efficiency of the assay ranged from 95% to 100%, all the assays were carried out simultaneously for *LsMTP* and *ef1α* using the same cDNA and master mix along with two negative controls, a nontemplate control and a no reverse transcriptase control. All the samples were run in duplicate, and Ct (cycle threshold) values were averaged. The final results were analyzed using the 2^−ΔΔCT^ method (27). The Q-PCR analysis was performed on lice recovered from two RNAi experiments. Primers used in Q-PCR for the detection of downregulation in the RNAi experiments were designed outside the double-stranded RNA (dsRNA) fragments. For each RNAi experiment, five representative adult females from the control group and *LsMTP* dsRNA-treated group were analyzed. Animals from the control group were used as a calibrator to calculate relative expression. Relative expression levels of three variants of *LsMTP* in different developmental stages of salmon lice were also determined by Q-PCR using copepodids as a calibrator. For the starvation experiment, animals (n = 5) were collected on days 0, 1, 2, and 4, and after 2 days of refeeding on the host fish. Relative expression of *LsMTP* was calculated using day 4 for calibration.

### RNAi

dsRNA was prepared according to the Megascript RNAi kit (Ambion). Two different fragments targeting different regions of *LsMTP* mRNA (Fig. 1) were amplified by PCR from primers with T7 promoter sequence, previously used for synthesis of in situ hybridization probes (supplemental Table S1). A fragment of 850 bp from cod trypsin (*CPY185*) was used as a control (23). Respective PCR products were used as templates for the synthesis of sense and antisense RNAs by in vitro transcription using T7 polymerase. For synthesis of dsRNA sense and antisense, RNAs were pooled and incubated at 75°C for 5 min followed by slow cooling to room temperature. The purified dsRNA concentrations were measured with Nanodrop ND 1000 Spectrophotometer, and a final concentration of 600 ng/μl was used for injections.

Two RNAi experiments were conducted separately in female lice. The first knockdown of *LsMTP* was carried out with dsRNA fragment 1 (LsMTP Fr 1) in newly molted preadult II females. In the second experiment, *LsMTP* silencing was done with dsRNA fragment 2 (LsMTP Fr 2) in young adult female lice. Both RNAi experiments were performed as described by Dalvin et al. (23). In each experiment, female lice were injected for *LsMTP* dsRNA and control dsRNA separately. After injection of dsRNA, lice were kept in seawater for 3 h and put back (n = 30–32) on three fish for every dsRNA fragment with equal numbers of dsRNA-treated female and untreated male lice. Both RNAi experiments were terminated when control dsRNA-injected female lice produced second pairs of egg-strings. Female lice were examined for gross morphology and imaged along with egg-strings for further egg-string measurement. Afterwards, egg-strings from females of both experiments were removed gently with forceps, placed into individual hatching incubators and closely examined every day. The offspring from the first RNAi experiment were evaluated visually and counted at 9 days post hatching when control animals had developed to copepodids. Nauplii from the second RNAi experiment were collected, visually evaluated, and counted between 6 and 8 h post hatching and closely followed through molting to nauplii II and further to copepodids. Neutral lipid content was detected and quantified in nauplii I using lipid stains (see below).

### Lipid analysis

Seven independent replicates of groups of 25 nauplii hatched from control and LsMTP dsRNA-injected female egg-strings from the second RNAi experiment were used for qualitative and semi-quantitative analysis of neutral lipids.

#### Oil Red O staining.

Nauplii I were collected from hatching incubators, washed three times with cold PBS, and fixed in phosphate-buffered 4% paraformaldehyde (pH 7.4) for 2 h. Oil Red O stain was performed using the methods described (28) with some modifications. Fixed nauplii were washed three times with cold PBS, resuspended in 60% isopropanol for 10 min, and stained with Oil Red O stain (Sigma-Aldrich) for 30 min. After staining, nauplii were washed in cold PBS, rinsed with 60% isopropanol, mounted, and photographed with a Leica Model MZ6 stereo microscope.

#### Nile Red staining.

Nile Red stain was used to detect neutral lipids in unfixed nauplii I according to (29) with the following modifications. Nauplii I were washed three times with cold PBS, stained with 1 ug/ml of Nile Red (Sigma-Aldrich) in PBS for 30 min, and imaged directly with a Leica TCS SP5 confocal microscope. Neutral lipids were visualized by excitation at 543 nm and fluorescence detection at 635 nm.

#### Semi-quantification of total nauplii I neutral lipids.

The semi-quantification of total neutral lipids of nauplii I was carried out using Oil Red O stain. After fixation and staining with Oil Red O, the excess stain was washed away with 60% isopropanol. Oil Red O stain was extracted from nauplii using 200 μl of 100% isopropanol and absorbance was measured at 500 nm in duplicate. Background signal was subtracted using 100% isopropanol as a background control.

### Bioinformatics analysis

The Staden package (30) was used for DNA sequence assembly, editing, and analysis. Multiple sequence alignment was done in BioEdit version 7.2.5 (31) using ClustalW. Accession numbers of MTP protein sequences from other species included in the multiple alignment were as follows: *Homo sapiens* (NCBI: NP_000244.2), *Salmo salar* (NCBI: XP_014050992.1), *Danio rerio* (NCBI: NP_998135.1), *D. melanogaster* (NCBI: NP_610075.2), *Zootermopsis nevadensis* (NCBI: KDR21635.1), *Daphnia magna* (NCBI: JAN30039.1), *Scylla olivacea* (Uniport: A0A0P4WDH4), and *Xenopus tropicalis* (NCBI: XP_002934813.1). Signal peptides were predicted using Phobius (http://phobius.sbc.su.se/) and SignalP server (http://www.cbs.dtu.dk/services/SignalP/). Conserved domain (LpD-N) in the protein sequences was analyzed in Conserved Domain Database (32). Secondary structures of proteins were predicted using JPred4 (33) or PSSpred (http://zhanglab.ccmb.med.umich.edu/PSSpred/) and three-dimensional structures of proteins were resolved using Phyre2 online server (34). All predicted structures of proteins were refined using Modrefiner (35) and visualized using Pymol software.

## RESULTS

### *LsMTP* gene encodes three transcript variants

One DNA sequence encoding *LsMTP* was identified in the salmon louse genome. To obtain the full-length transcript sequence, 5′ and 3′ RACE were carried out using primers specific to the *LsMTP* sequence, which revealed three transcript variants named *LsMTP-A*, *LsMTP-B*, and *LsMTP-C* (**Fig. 1A**). Variant *LsMTP-A* contained alternative exon 1, which was part of the 5′ untranslated region (UTR) sequence in variant *LsMTP-B*, but not present in variant *LsMTP-C* (Fig. 1A, B). Predicted open reading frames of *LsMTP-B* and *LsMTP-C* variants, both started from the alternative start codon located in the intronic region of variant *LsMTP-A* (Fig. 1A, B). The details of the all LsMTP variants are summarized in Fig. 1D.

### Sequence and structural analysis of *LsMTP* variants

The *LsMTP-B* and *LsMTP-C* transcripts encoded an identical protein of 819 amino acids, while the LsMTP-A was 827 amino acids and contained a different N-terminal signal peptide (Fig. 1C). The LsMTP-A and LsMTP-B/C isoforms contained a conserved region, named the lipoprotein N-terminal domain (SMART accession SM00638), found in lipid transport proteins, including vitellogenins, apoLp, and apoB. A BLASTP search in the UniProtKB/Swiss-Prot revealed LsMTP as most closely related to an uncharacterized protein from the crab *Scylla olivacea* (25% identity) and MTP of *Daphnia magna* (23% identity). A similar identity of LsMTP was also found with other functionally known MTPs like *Homo sapiens* (21.8%), *Mus musculus* (21.1%), *Danio rerio* (21.8%), *Gallus gallus* (21.0%), and *D. melanogaster* (21.7%). Alignment to MTP of its host, the Atlantic salmon (NCBI: XP_014050992.1) showed 22% identity to LsMTP.

**Fig. 2.**
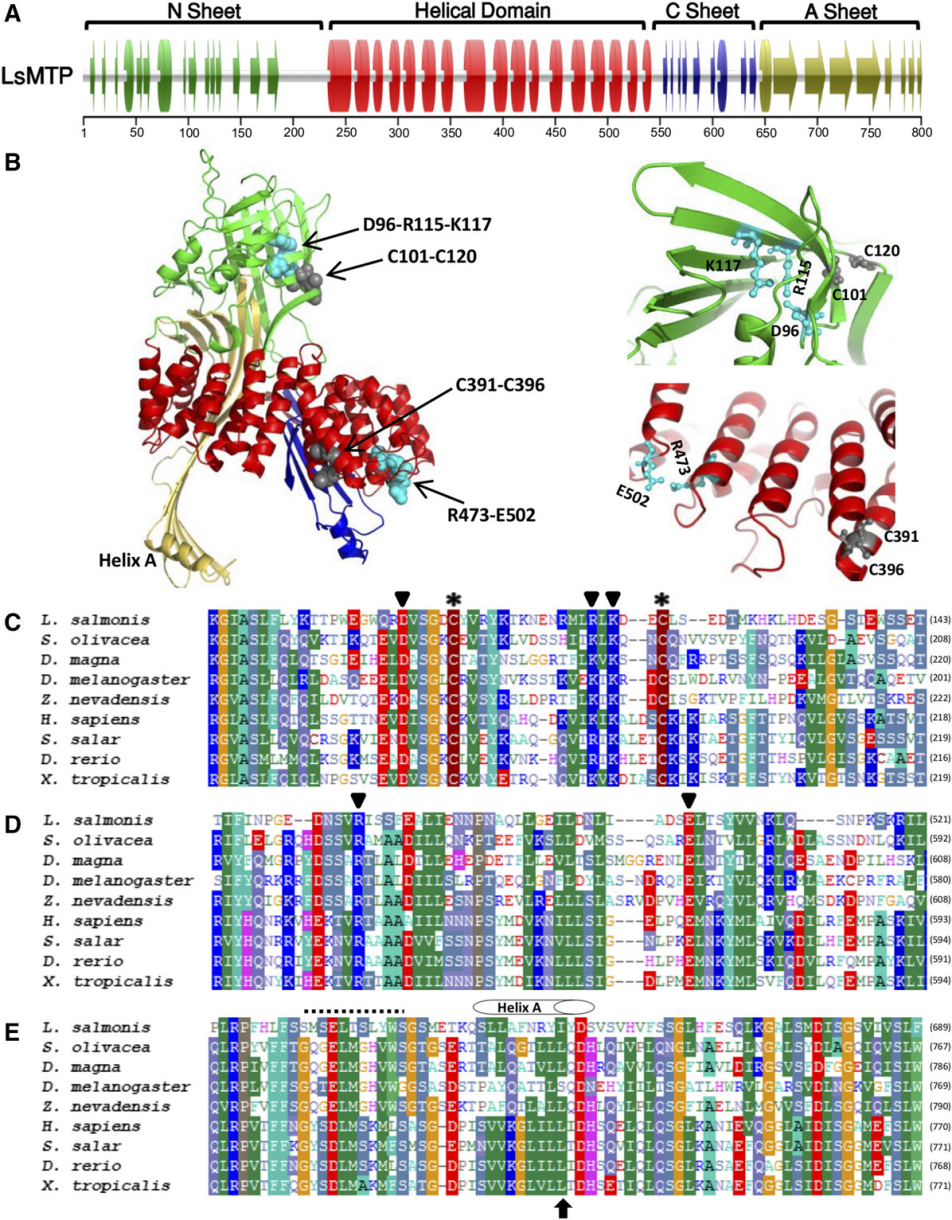
Structural analysis of LsMTP. A: Predicted secondary structures of LsMTP. LsMTP model consists of four functional domains: N-terminal β-sheet, central helical domain, C β-sheets, and A β-sheets. The ruler for amino-acid numbering is shown below. B: The tertiary structure of LsMTP was modeled using PHYRE protein structure prediction program. The left panel represents the full view of the ribbon structure of LsMTP protein, with cysteine residues (gray spheres) and residues of the salt bridges (cyan spheres). The right panel represents the zoom view of the interior of the N-terminal β-sheet (upper) and central helical domain (lower). Disulfide linkages have been formed between C101-C120 and C156-C182, whereas salt bridges have been formed between D96-R115-K117 and R473-E502. C, D: Multiple alignments of the conserved N-sheet and central helical domain of LsMTP with other MTPs. The conserved cysteine residues are shown with asterisks and residues of the salt bridges are highlighted with inverted triangles. E: Multiple alignment of the MTP-specific sequence. This region was present in salmon louse MTP and contained helix A. The black arrow below the sequence indicates the amino acid position (L734 in human MTP) important for the lipid transfer activity (37). Isoleucine (I653) was found at this position in salmon lice. The dotted line shows the helix as predicted by Jpred4 and PSSpred, which has not been described before in other MTPs.

Secondary and tertiary structures of the mature LsMTP (without signal peptide) were modeled along with other MTP orthologs (human, *Drosophila*, frog, and worm) using a homology modeling approach. The modeling template was the X-ray crystal structure of the silver lamprey (*Ichthyomyzon unicuspis*) lipovitellin, the mature form of vitellogenin (PDB ID: 1LSH) (36), also used for domain database annotation and in similar modeling studies (37, 38). All the modeled structures (**Fig. 2**, supplemental Fig. S1) displayed similar domain composition made up of an N-terminal β-barrel (N-sheet), a central helical domain, and two β-sheets (C-sheet and A-sheet) toward the C terminal, in agreement with the lipovitellin template. Moreover, structural similarity between the MTPs was calculated by performing structural alignments in PyMol with salmon louse MTP structure as a reference. The average distance between the atoms of the superimposed proteins, calculated as root mean square deviation (RMSD), was found to be 2.52, 3.07, 3.07, and 10.2 Å for *H. sapiens*, *D. melanogaster*, *X. tropicalis*, and *C. briggsae*, respectively.

In the N-sheet, the salmon louse MTP model predicted 13 antiparallel β-strands (Fig. 2A, B), similar to the available lipovitellin structure where 11 of the 13 β-strands formed a barrel-like conformation (9). The central helical domain of the salmon louse MTP model consisted of 17 α-helices arranged in an inner and outer layer (Fig. 2A, B), as in the lipovitellin structure. The C-sheet domain in the lipovitellin X-ray structure contained two β-sheets (C and A), which were also found in the sea louse MTP model. The two β-sheets formed a hydrophobic pocket (Fig. 2A), which included a number of hydrophobic residues (37). Sequence alignment of the conserved N-sheet, the central helical domain, and the C-sheet of MTP from salmon lice, crustaceans, vertebrates, and insects showed that the two cysteines known to form a disulfide bond in the N-sheet domain of lamprey lipovitellin (9) were conserved (C156-C182 in lipovitellin, C101-C120 in LsMTP) (Fig. 2B, C). This disulfide linkage in the N-sheet domain was essential to stabilize the barrel-like conformation formed by the β-strands. Walsh et al. (39) found an amino acid (D169) in the N-sheet domain, which was important to the formation of an internal salt bridge with amino acids K187 and K189. Missense mutation (D169V) destroyed this salt bridge, which led to loss of PDI binding as well as lipid transfer activity. In the N-sheet domain of LsMTP, amino acid D96 formed an internal salt bridge with amino acids R115 and K117 (Fig. 2B).

The central helical structure in lipovitellin was stabilized by a disulfide linkage (C451-C486 in lamprey lipovitellin, C391-C396 in LsMTP) (Fig. 2A). The residues in the salt bridge (R547-E574 in lamprey lipovitellin, R473-E502 in LsMTP) in the helical domain of lipovitellin were also conserved in the LsMTPs (Fig. 2D). In lipovitellin, this salt bridge was important to tie together helices 14 and 16 and increased the stability of the local fold. Further, the MTP-specific sequence (Fig. 2E), which was not present in apoB, apoLp, vitellogenin, phospholipid transfer proteins, and other lipid transfer proteins (40), was also conserved in the salmon louse MTP. Two helixes (helix-A and helix-B) in the A-sheet of human MTP were known to be involved in lipid transfer activity (37). The amino acid, leucine-734, in helix-A was important in lipid transfer and conserved in vertebrates. However, in helix A (A-sheet) of LsMTP, isoleucine (I653) was found at this position (Fig. 2E).

### Tissue distribution of *LsMTP* transcripts in the adult female lice

**Fig. 3.**
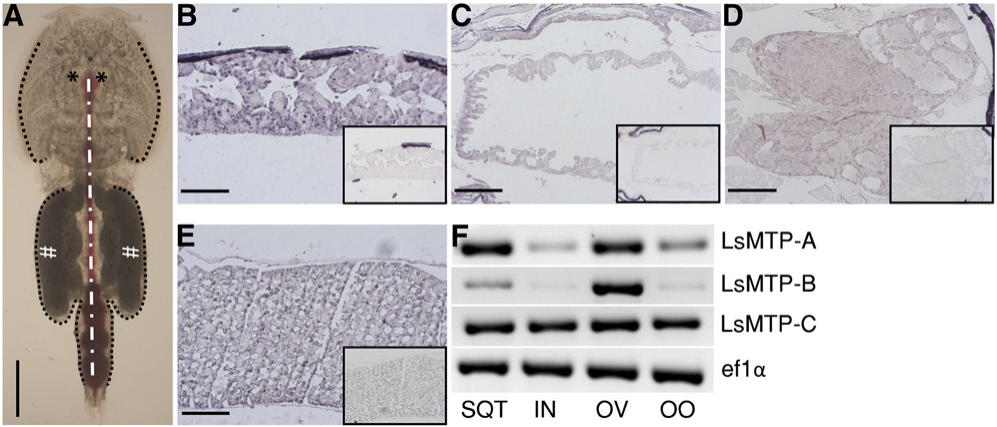
In situ hybridization and RT-PCR analysis of *LsMTP* mRNAs in various tissues of an adult female salmon louse. A: Dorsal view of an adult female without egg-strings. The black dotted line indicates the area where sub-cuticular tissue is situated; a white straight dash-dot line represents the gut filled with blood. Asterisks (*) and hashtags (#) represent the positions of the ovaries and mature vitellogenic oocytes, respectively. B–E: Cross-sections of sub-cuticular tissue (B), intestine (C), ovaries (D), and vitellogenic oocytes (E) hybridized with antisense probes or sense probes (small inserts) as negative controls. F: RT-PCR analyses from cDNA templates of different tissues of adult female lice using *LsMTP* variant-specific primers. RT-PCR analysis of *ef1α* was carried out to determine the quantitative variations of *LsMTP* transcripts among samples. SQT, sub-cuticular tissue; IN, intestine; OV, ovaries; OO, oocytes. Scale bars = 1 mm (A), 200 μm (B, E), 100 μm (C, D).

In order to localize the site of expression of *LsMTP* in the adult female louse, in situ hybridization was performed on sections. Two independent probes targeting different parts of the transcript detecting all the three variants of *LsMTP* were utilized. The two probes revealed the same localization pattern of *LsMTP* transcripts in the female (**Fig. 3B–E**). *LsMTP* transcripts were detected in the sub-cuticular tissue (Fig. 3B), intestine (Fig. 3C), ovaries (Fig. 3D), and vitellogenic oocytes in the genital segment (Fig. 3E). No positive signal was detected in slides treated with sense probes (negative control).

### Differential expression of *LsMTP* transcript variants in different tissues by RT-PCR

RT-PCR with variant-specific primers (supplemental Table S1) showed a differential expression of *LsMTP* variants among the examined tissues. *LsMTP-A* was mainly expressed in the sub-cuticular tissue and ovaries (Fig. 3F). *LsMTP-B* was detected in the ovaries, with relatively low abundance in the sub-cuticular tissue, while *LsMTP-C* was present in sub-cuticular tissue, intestine, ovaries, and vitellogenic oocytes (Fig. 3F).

### Expression level of *LsMTP* variants in different developmental stages

**Fig. 4.**
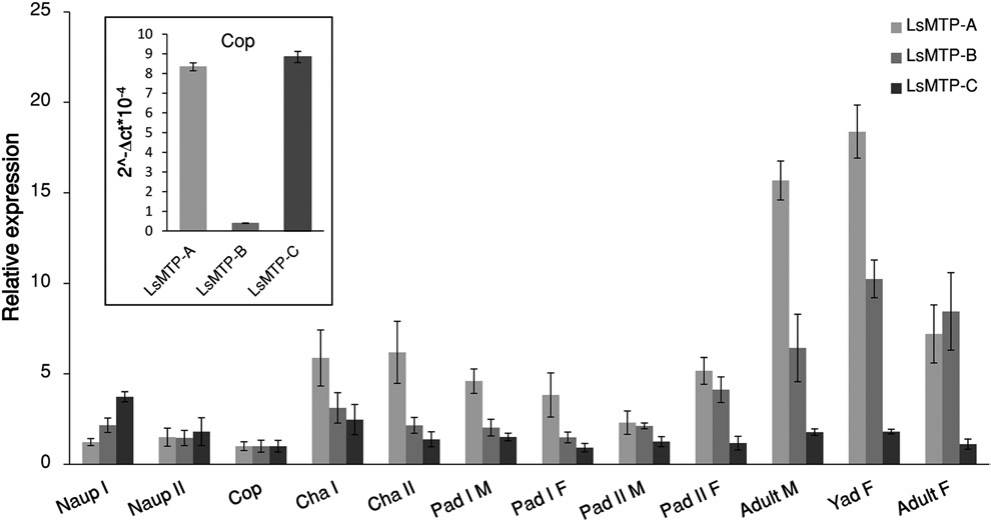
Expression of the transcript levels of three different variants of *LsMTP* in various developmental stages of the salmon louse relative to transcript level in the copepodids. The insert shows the expression of the three variants in the copepodids. Naup I, nauplii I; Naup II, nauplii II; Cop, planktonic copepodids; Cha I, chalimus I; Cha II, chalimus II; Pad I M, preadult I male; Pad I F, preadult I female; Pad II M, preadult II male; Pad II F, preadult II female; YAD, young adult female. Error bars represent the SD (n = 5 for each stage).

Analysis of mRNA levels using Q-PCR showed that all three splice variants of *LsMTP* were expressed at all developmental stages of salmon lice (**Fig. 4**). Expression levels of variants *LsMTP-A* and *LsMTP-B* were highest in adult male and female stages as compared with other stages (Fig. 4). However, relative expression of *LsMTP-A* was reduced in the adult female when compared with the young adult female (newly molted females) and in the adult male, the expression of *LsMTP-B* was relatively higher than *LsMTP-A*. Moreover, the expression of *LsMTP-C* was relatively stable, with the highest expression levels found in nauplius I (Fig. 4).

### Knockdown of *LsMTP* gene inhibits egg production and reduces larva survival

**Fig. 5.**
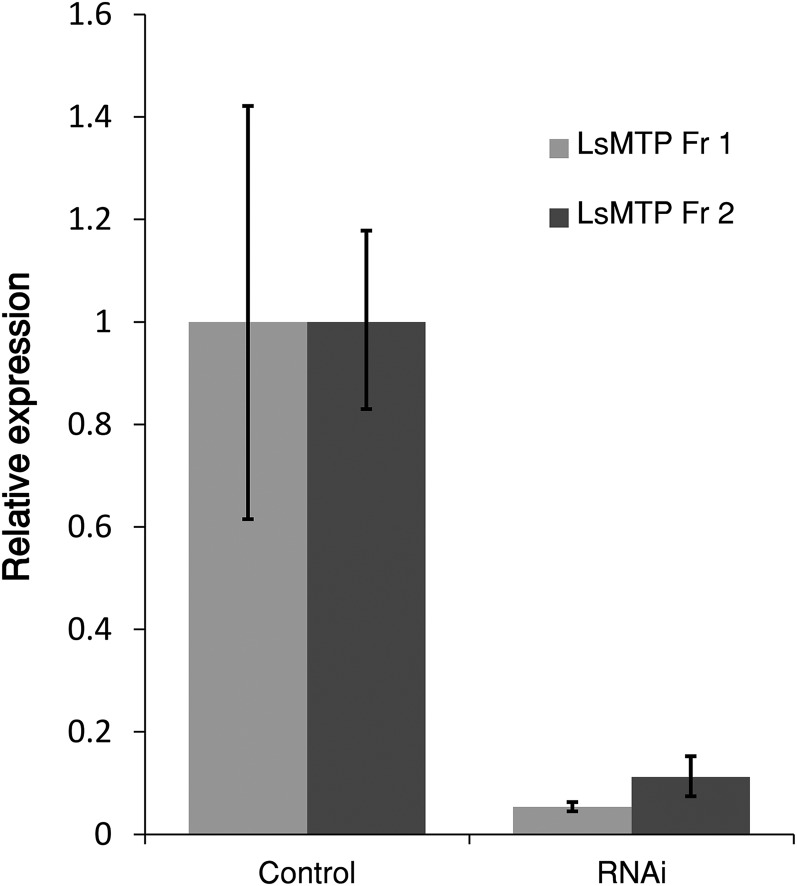
Inhibition of *LsMTP* transcript by RNAi in adult female salmon lice. The expression level of *LsMTP* was quantified by Q-PCR in adult females injected with dsRNA in preadult (Fr 1) and young adult (Fr 2) females against control. The results represent the mean ± SEM of five biological replicates from each treatment group. Significant downregulation of *LsMTP* was found as compared with control (*t*-test, *P* < 0.05).

Two experiments with RNAi knockdown of *LsMTP* were performed, one in newly molted preadult II females (n = 30) and a second in young adult females, using dsRNA targeting cod trypsin in the negative control groups (**Table 1**). Both RNAi experiments were terminated when the adult females from the control groups produced the second pair of egg-strings to be certain that maturing eggs received dsRNA treatment. In the first experiment, RNAi knockdown of *LsMTP* in newly molted preadult II females (n = 30), the downregulation of *LsMTP* was highly significant and the levels of *LsMTP* were reduced by 95% compared with control animals (**Fig. 5**). The knockdown of *LsMTP* had no lethal effect on the adult lice. However, females injected with *LsMTP* dsRNA produced shorter and curly egg-strings compared with control females, which produced normal straight egg-strings, (supplemental Fig. S2). A very strong reduction (90%) in the number of surviving copepodids produced from the females treated with *LsMTP* dsRNA was observed, as compared with control groups (Table 1). The few larvae that hatched went through molting, apparently as normal, and developed into copepodids. This low number of surviving larvae could be caused by reduced lipid deposition in the developing oocytes, while still sufficient for some larvae to survive. To address this possibility and to further confirm the specificity of RNAi-mediated gene silencing, a second RNAi was performed in newly molted young adult females (n = 32) that should give higher initial MTP transcript level, and therefore somewhat higher survival rate of the larvae. Downregulation of *LsMTP* gene expression was approximately 90% compared with control (Fig. 5), with the remaining absolute levels 2-fold higher than in the first RNAi experiment. All animals in the control group produced normal egg-strings, while about 50% (11 females) of the RNAi-treated females still produced egg-strings. These egg-strings were short and curly when compared with egg-strings of control females (supplemental Fig. S3). Furthermore, a significant number (∼72%) of hatched nauplii from *LsMTP*-treated females did not develop to copepodids when compared with the control group (Table 1).

**TABLE 1.** Summary of the RNAi experiments

	Injected Female Lice	Recovered Female Lice	Number of Females that Produced Egg-Strings	Length of Egg-Strings (mm)	Number of Hatched Nauplii	Number of Hatched Copepodids
Experiment 1						
Control	30	12	12	18.8 ± 1.6 (n = 9)	Not counted	322 ± 109 (n = 9)
RNAi (fragment 1)	30	10	7	7.4 ± 3.6 (n = 6)	Not counted	33 ± 16 (n = 5)
Experiment 2						
Control	30	23	23	18.4 ± 1.8 (n = 20)	348 ± 78 (n = 7)	314 ± 67 (n = 7)
RNAi (fragment 2)	32	22	11	15.5 ± 3.8 (n = 7)	262 ± 97 (n = 6)	86 ± 61 (n = 6)

**Fig. 6.**
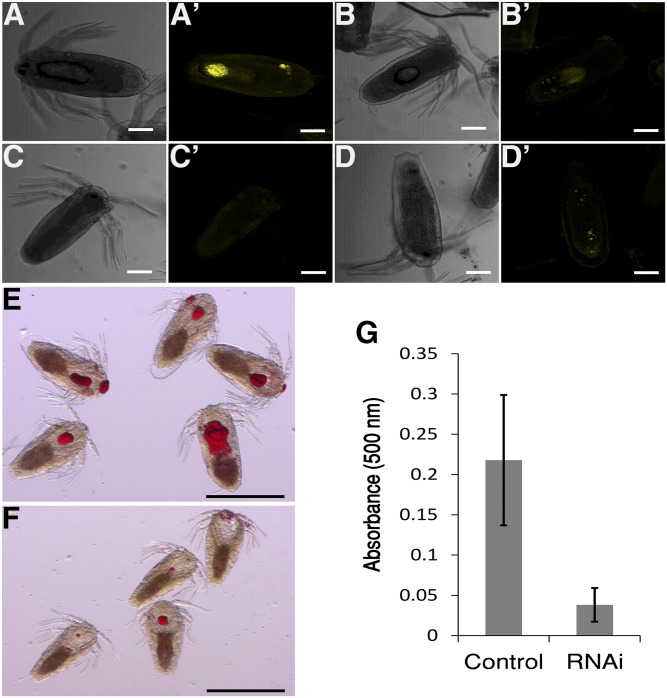
LsMTP transports maternal neutral lipids to developing embryos. Bright field (A–D) and confocal fluorescence (A′–D′) nauplii stained with Nile Red to visualize the lipid droplets. Accumulation of maternal neutral lipids was reduced in the nauplii of females injected with *LsMTP* dsRNA (C′–D′) as compared with nauplii produced from females injected with control dsRNA (A′–B′). Nauplii hatched from females injected with control dsRNA (E) and *LsMTP* dsRNA (F) were stained with a nonfluorescent dye (Oil Red O). Nauplii hatched from females injected with *LsMTP* dsRNA accumulate fewer neutral lipids (F), as compared with nauplii hatched from females treated with control dsRNA (E). G: Semi-quantification of neutral lipids with Oil Red O stain in the nauplii of females treated with control and *LsMTP* dsRNAs. Neutral lipids were reduced significantly (83%) in hatched nauplii of *LsMTP* dsRNA-treated females. Results are represented as the mean ± SD of nauplii (n = 25) hatched from seven independent replicates of control and *LsMTP* dsRNA-injected females. Scale bars = 100 μm (A/A′–D/D′), 500 μm (E, F).

To explore the level of lipids in larvae from controls and RNAi knockdown animals, nauplii hatched from the egg-strings were stained to detect neutral lipids with Nile Red and Oil Red O. Similar results were obtained with the two different stains. Nauplii produced from females injected with *LsMTP* dsRNA had no or fewer lipid droplets in their yolk as compared with nauplii produced from females injected with control dsRNA (**Fig. 6A–F**). Total neutral lipids were measured using Oil Red O in hatched nauplii of *LsMTP* and control RNAi-treated females. As expected, a significant reduction (83%) of total neutral lipids was seen in nauplii of *LsMTP* RNAi-treated females (Fig. 6G).

### Starvation reduces the LsMTP mRNA level in adult female lice

**Fig. 7.**
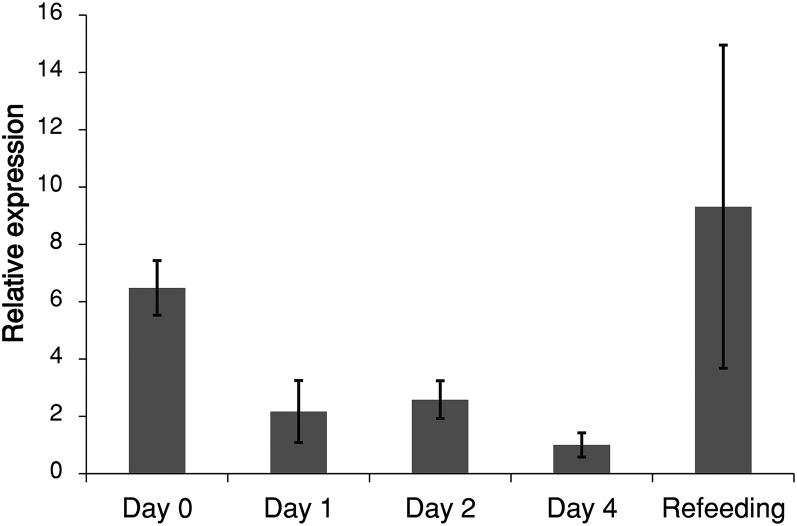
Starvation reduces *LsMTP* transcript levels in adult female lice. Significant reduction of the expression level of *LsMTP* was found in starved animals, as compared with control animals (day 0) (*t*-test: *P* < 0.05). Refeeding (2 days) increased the expression of LsMTP as compared with starved and control (day 0) animals. Error bars represent the SD for each time point (n = 5).

Adult female lice obtain important nutrients, such as lipids, from the fish host’s blood and skin. To determine whether *LsMTP* mRNA expression was directly correlated with food uptake, adult female lice were starved by removal from the host, and then refed on the fish host. Samples for Q-PCR analysis were collected at 0 (control), 1, 2, and 4 days, and 2 days of refeeding. The expression of *LsMTP* was reduced 70–85% following starvation and increased upon refeeding (**Fig. 7**).

## DISCUSSION

MTP has been studied in many organisms, particularly in vertebrates, due to its important role in lipid metabolism. Three transcript variants of *LsMTP*, referred to as *LsMTP-A*, *LsMTP-B*, and *LsMTP-C* (Fig. 1), were identified. *LsMTP-A* mRNA is predicted to encode an 827 amino acid protein, whereas *LsMTP-B* and *LsMTP-C* encode an 819 amino acid long protein (LsMTP-B/C). Two isoforms of MTP (MTP-A and MTP-B) have been identified in mice due to alternative first exons, and both isoforms are effective in lipid transfer activity (41, 42). Recently, two splice variants (*MTP-B* and *MTP-C*) have been found in humans (43), and it has been concluded that alternative splicing and the presence of distinct promoter regions play a key role in the regulation of cellular MTP levels. Additionally, specific 5′ UTRs containing elements that alter translation enable the cell to optimize MTP activity. The predicted protein isoforms of *L. salmonis* have only 21% to 23% sequence identity to MTPs of other species. Similarly, MTP proteins from other invertebrates (insects and nematodes) have been reported to have less than 25% sequence identity compared with human and zebrafish (15, 40). Therefore, a considerable identity difference is present between the MTPs of invertebrates and vertebrates.

Further structural analysis predicted that LsMTP protein contains β-sheets (N, C, and A) and a central α-helix C-terminal domain (Fig. 2). Similar structural domains have also been found in MTP of *H. sapiens*, as well as in other MTP orthologs such as *D. rerio*, *D. melanogaster*, *C. elegans* (40), and *M. amblycephala* (44). The RMSD was found to be 2.52, 3.07, 3.07, and 10.2 Å for *H. sapiens*, *D. melanogaster*,* X. tropicalis*, and *C. briggsae*, respectively. The high RMSD found for *C. briggsae* could be connected with lower numbers of β-strands in the N-terminal domain (supplemental Fig. S1). Of the other protein sequences tested, the results indicate a significant overall structural similarity.

*LsMTP* transcripts were found in the sub-cuticular tissue (tissue with a functional resemblance to insect fat body and vertebrate liver) and intestine. In the salmon louse, the sub-cuticular tissue is the site where two vitellogenins (21) and yolk-associated protein (23) are produced. In *C.* *elegans*, *MTP* transcripts were found in the intestine, which is reported to function as a secretory organ of vitellogenins (16). Similarly, in *D. melanogaster*, *MTP* transcripts were found in the fat body (an organ analog to vertebrate adipose tissue and liver), and MTP proteins are reported to promote the production of Lpp and large lipid transfer particles (14). *MTP* transcripts were also found in vertebrates in tissues expressing apoB, such as liver, intestine, retina (45), kidney (46), myocardium (47), placenta, and yolk sac (48–50). Here, MTP functions as an essential chaperone for the assembly and secretion of apoB-lipoproteins. *LsMTP* transcripts were also found in the ovaries and oocytes of the salmon louse. In vertebrates, the *MTP* is found in the ovaries and testis, where *apoB* is not expressed (51). The function of MTP in these lipid-affluent tissues is unknown. However, it is likely that MTP may play an important role in lipid trafficking and/or storage (52, 53). Further studies are needed to demonstrate the tissue-specific function of LsMTP in the salmon louse.

Expression levels of the three *LsMTP* transcript variants were also analyzed in some key adult female louse tissues (Fig. 3F). The observed differences between expression of the three *LsMTP* variants in different tissues of adult female lice are similar to observations in vertebrates, such as mice and humans (41–43). For example, mice have two isoforms of *MTP*. The *MTP-A* isoform is expressed predominantly in the liver, intestine, and heart, whereas *MTP-B* is mainly found in adipose tissue. Similarly, in humans, splice variant *MTP-B* is found in various tissues, whereas *MTP-C* is expressed mainly in brain and testis.

In copepodids, the *LsMTP-A* and *LsMTP-C* transcripts are clearly the most abundant forms (Fig. 4, insert). Assessing the *LsMTP* transcript levels throughout the *L. salmonis* life cycle, relative to copepodids, showed that *LsMTP-A* and *LsMTP-B* forms varied with highest levels in adult lice of both sexes, whereas *LsMTP-C* was relatively stably transcribed. The salmon louse larval stages are nonfeeding and survive on maternally provided energy, whereas the parasitic stages have access to a stable and abundant amount of energy. The steep increase in the *LsMTP-A* variant (and to some extent the *LsMTP-B* variant) in adults probably reflects increased demand of lipids for gamete production, particularly in females, because these forms are expressed in the sub-cuticular tissue and ovary (Fig. 3F). Our data indicate that, in the intestine, *LsMTP-C* is the most abundant form (Fig. 3F) and have a stable expression levels in the various developmental stages (Fig. 4). These results indicate that *LsMTP-C* is involved in lipoprotein maturation and secretion from intestinal cells and that increased intestinal capacity is a result of growth (i.e., increased number of enterocytes).

To investigate the effect of starvation, adult females were removed from their hosts. A significant reduction in expression of *LsMTP* was seen over time and refeeding increased the expression, as compared with starved and control (day 0) animals. This indicates that expression of *LsMTP* directly depends on the availability of food in the intestine and/or lipids in the blood-feed, or is affected indirectly due to downregulation of other lipid-carrying lipoproteins. The effect of feeding on *MTP* mRNA expression has been studied in *D. rerio* and a significant pretranslational increase in *MTP* expression was seen in the anterior intestine (54). In mammals, in contrast, *MTP* mRNA expression was not significantly changed in the intestine and liver due to fasting, but a moderate pretranslational increase was noted because of high fat and cholesterol diets (55–58).

LsMTP is expected to play an important role in the transport of yolk lipids from the intestine to growing oocytes through the secretion of lipoproteins. The results of RNAi in young adult females clearly showed that the larvae of *LsMTP* knockdown female lice had a significantly lower reserve of maternally deposited lipids in their yolk, as confirmed by our qualitative and quantitative lipid analysis (Fig. 6A–G). Furthermore, a significant reduction in the number of copepodids was also noted (Table 1). To our knowledge, no information about the function of MTP is available in other crustaceans. However, knockout of the *MTP* gene in homozygous mice was embryonic lethal, due to the failure of the yolk sac to deliver lipids to the developing embryos (12). Similarly, disruption of *dsc-4*, a homolog of *MTP* in *C. elegans*, by RNAi or mutation, suppresses the germline delay and egg-laying (16). Moreover, MTP has also been shown to be important for the secretion of Lpp and large lipid transfer particles in the hemolymph of *D. melanogaster* and *MTP* mutant larvae; neutral lipid accumulates in the gut due to loss of lipoproteins (14). MTP is also important for yolk lipid utilization and absorption of dietary neutral lipids in larvae of *D. rerio* (59). The significant reduction in egg production and mortality of copepodids suggests that LsMTP has an important function in the reproduction and lipid metabolism of salmon lice. The specific mechanism for the reduction of lipids in the embryos of the *LsMTP* knockdown female is not known. However, it can be suggested that LsMTP is essential for the secretion of lipoproteins, similar to mammalian lipoproteins, and serves as a vessel for the transport of lipids from the intestine to oocytes or other tissues of the salmon louse.

In summary, we identified *MTP* in the salmon louse, and structural analysis revealed that LsMTP has similar functional domains found in MTP homologs from other species. From results of expression analysis together with functional studies, it can be concluded that LsMTP has an important role in the lipid metabolism and reproduction of salmon lice. Results from our study further demonstrated that LsMTP could be used as a target for the control of reproduction in the female lice. However, further investigations are required to characterize associated lipoproteins and mechanisms of lipid loading and secretions in salmon lice.

## Supplementary Material

Supplemental Data
