# Supplementary material for: Microsomal triglyceride transfer protein in the ectoparasitic crustacean salmon louse (Lepeophtheirus salmonis)
Source: J Lipid Res. 2017 Jun 10;58(8):1613–23. doi: 10.1194/jlr.M076430 (PMC5538283; doi:10.1194/jlr.M076430)
Supplement: Supplemental Data [file supp_58_8_1613__index.html]

Microsomal Triglyceride Transfer Protein (MTP) in the ectoparasitic crustacean salmon louse (L. salmonis). — Microsomal triglyceride transfer protein in the ectoparasitic crustacean salmon louse (Lepeophtheirus salmonis) — Supplemental Data 

# Microsomal triglyceride transfer protein in the ectoparasitic crustacean salmon louse (*Lepeophtheirus salmonis*)

## Supplemental Data

- Supplement to JRL 2017 076430 (.pdf, 1.1 MB) - Supplemental table with primers used. Supplemental figure 1, structure of MTP. Supplemental figure 2, phenotype of RNAi treatment of pre-adult. Supplemental figure 3, phenotype of RNAi treatment of young adults.
