# Supplementary material for: Microsomal triglyceride transfer protein in the ectoparasitic crustacean salmon louse (Lepeophtheirus salmonis)
Source: J Lipid Res. 2017 Jun 10;58(8):1613–23. doi: 10.1194/jlr.M076430 (PMC5538283; doi:10.1194/jlr.M076430)
Supplement: Supplemental Data [file 10.1194_M076430_jlr.M076430-1.pdf]

SUPPLEMENTAL INFORMATION:

Microsomal Triglyceride Transfer Protein (MTP) in the ectoparasitic crustacean salmon louse (*L. salmonis*).

Authors

Muhammad Tanveer Khan[1], Sussie Dalvin[2], Frank Nilsen[1] and Rune Male[3,4]

1. Sea Lice Research Centre, Department of Biology, University of Bergen, P.O. Box 7803, N-5020 Bergen, Norway.
2. Sea Lice Research Centre, Institute of Marine Research, 5817 Bergen, Norway.
3. Sea Lice Research Centre, Department of Molecular Biology, University of Bergen, P.O. Box 7803, N-5020 Bergen, Norway.
4. Corresponding author.

Supplemental Table 1. List of Primers used in this study

Name	Sequence (5'-3')	Method/ Description
MTP_5RACE	GAACCGTTCGTTCGTGGGAGTGAGAGTATAGAGTA	5' RACE
MTP_3RACE	AGGAAGAGACCTTGATTTCAAATATCCATGAAGGC	3' RACE
M13_f	GTAAAACGACGGCCAG	Topo cloning
M13_r	CAGGAAACAGCTATGAC	Topo cloning
MTPF1	GCCTGGTTTGCCAATGTATC	PCR
MTPR1	CTCCACAGAAGATAGAGCCCA	PCR
MTPF2	TTGACGAGAAGACAGCAGTT	PCR
MTPR2	TGAGACTTCATTGCAGAGTTGAG	PCR
MTPp-FT7	GAAATTAATACGACTCACTATAGGGACGGTTCCTAG TGCATGTCT	In situ/dsRNA (Fragment 1)
MTPp-RT7	GAAATTAATACGACTCACTATAGGGCCACTGCATCT ATGACTTCCT	In situ/dsRNA
MTPp-F	ACGGTTCCTAGTGCATGTCT	In situ/dsRNA
MTPp-R	CCACTGCATCTATGACTTCCT	In situ/dsRNA
MTPp-F3 T7	TAATACGACTCACTATAGGGATGCTCAACTCCTCGG TGAA	In situ/dsRNA (Fragment 2)
MTPp-R3 T7	TAATACGACTCACTATAGGGGACACTATGACCGAGC	In situ/dsRNA

	CAGA	
MTPp-F3	ATGCTCAACTCCTCGGTGAA	In situ/dsRNA
MTPp-R3	GACACTATGACCGAGCCAGA	In situ/dsRNA
MTP_LE_SY_F	GGTCATACAGTTTCTGGTGAGACA	Q-PCR/RT-PCR (LsMTP-A)
MTP_LI_SY_F	GTGTCTTCGAGTGCGTGTTC	Q-PCR/RT-PCR (LsMTP-B)
MTP_S_SY_F	TGTCTGCACTACTCATAGAATGTTG	Q-PCR/RT-PCR (LsMTP-C)
MTP_LS_SY_R2	TCGTTCGTGGGAGTGAGAG	Q-PCR/RT-PCR (All variants)
MTP-SY-F	TGACGAGAAGACAGCAGTTAGT	Q-PCR (All variants)
MTP-SY-R	CTCCACAGAAGATAGAGCCCAA	Q-PCR (All variants)
qPCR EF1a fw	GGTCGACAGACGTACTGGTAAATCC	Q-PCR/EF1a
qPCR EF1a rv	TGCGGCCTTGGTGGTGGTTC	QPCR/EF1a

RACE, Rapid Amplification of cDNA: Ends, TOPO, DNA topoisomerase: Q-PCR, Quantitative real-time PCR: Insitu, Insitu hybridization: dsRNA, double-stranded RNA.

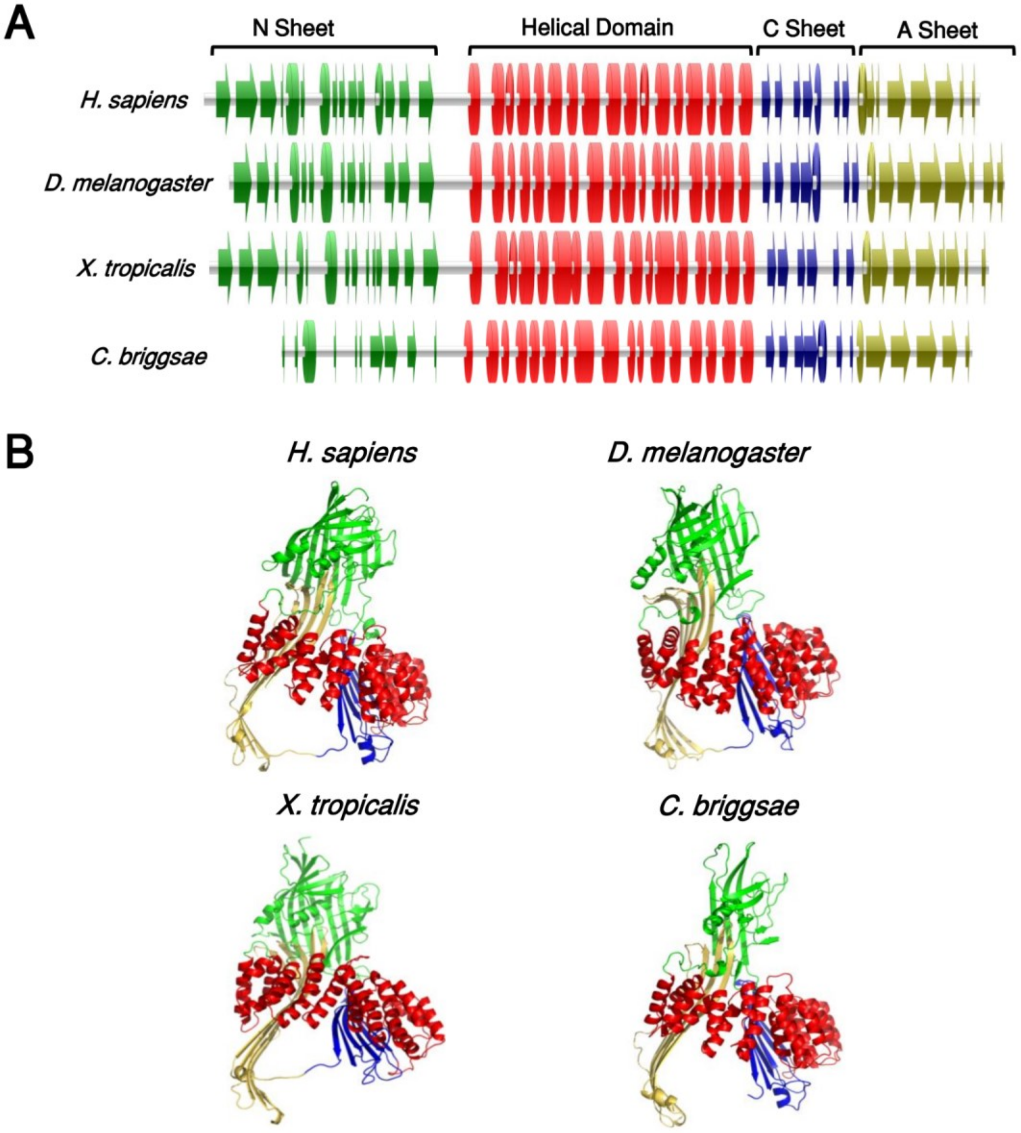

Supplemental Fig. 1: (A) Predicted secondary structures of the MTP of *Homo sapiens* (NCBI Reference Sequence: NP_000244.2), *Drosophila melanogaster* (NCBI Reference Sequence: NP_610075.2), *Xenopus tropicalis* (NCBI Reference Sequence: XP_002934813.1) and *Caenorhabditis briggsae* (NCBI Reference Sequence: XP_002634982.1). (B) Predicted 3D structures of the MTPs using Phyre (Protein Homology/analogy Recognition Engine V 2.0) webserver. Four structural domains were found in MTP orthologues: N-terminal β sheet, central helical domain, C β sheets and A β sheets.

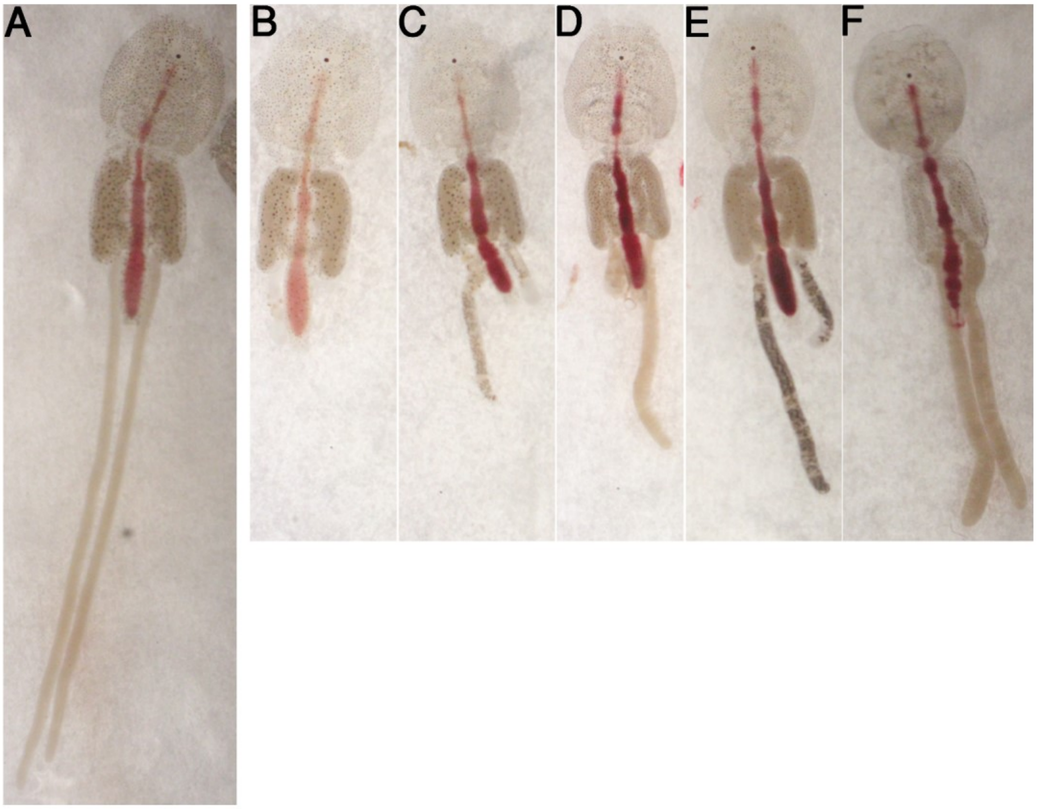

Supplemental Fig. 2: Representative phenotypes of the adult female lice egg-strings recovered after the *LsMTP* RNAi experiment using Fr. 1 in the pre-adult female lice. (A) Control animals with normal egg-strings (B-F) *LsMTP* dsRNA injected females has no and or short and abnormal egg-strings.

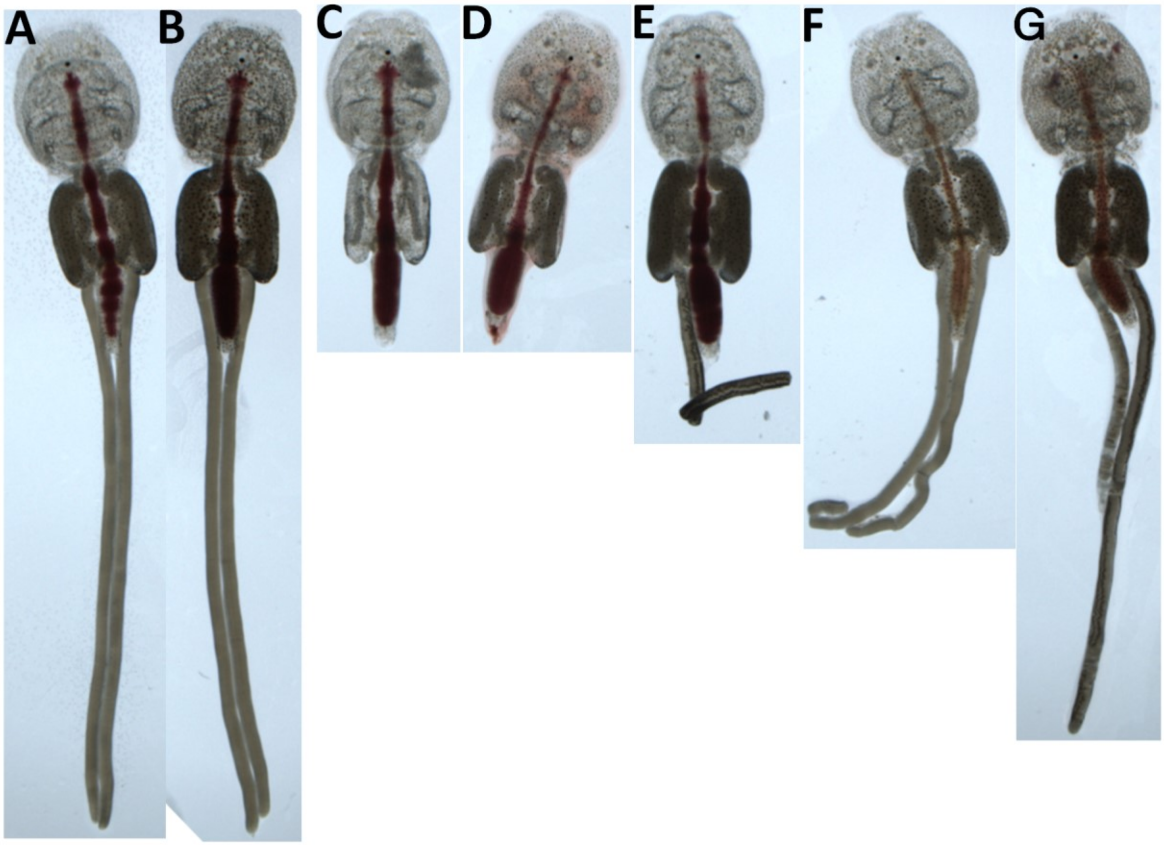

Supplemental Fig. 3: Representative phenotypes of adult female lice egg-strings obtained after the *LsMTP* RNAi conducted in young adult females using Fr. 2. (A-B) Control female lice with normal egg-strings (C-G) *LsMTP* injected female lice has no egg-strings or short and long abnormal egg-strings.
